# Immunoreactivity of the 14F7 Mab Raised against N-Glycolyl GM3 Ganglioside in Epithelial Malignant Tumors from Digestive System

**DOI:** 10.5402/2011/645641

**Published:** 2010-12-02

**Authors:** Rancés Blanco, Enrique Rengifo, Mercedes Cedeño, Charles E. Rengifo, Daniel F. Alonso, Adriana Carr

**Affiliations:** ^1^Department of Quality Control, Center of Molecular Immunology, Havana 11600, Cuba; ^2^Department of Pathology, Manuel Fajardo General Hospital, Havana 10400, Cuba; ^3^Laboratory of Molecular Oncology, Department of Science and Technology, Quilmes National University, Buenos Aires, Bernal B1876BXD, Argentina; ^4^Research and Development Direction, Center of Molecular Immunology, 216 Street and 15 Avenue Atabey, Playa. P.O. Box 16040, Havana 11600, Cuba

## Abstract

The limited expression of N-Glycolyl GM3 (NeuGcGM3) ganglioside in human normal tissues, as well as its presence in melanoma and breast carcinoma using 14F7 Mab (anti-NeuGcGM3), has been previously reported. In this work we evaluated for the first time the 14F7 Mab immunorecognition in some digestive system tumors. Immunohistochemical assays were made with 14F7, followed by anti-mouse biotinylated antibody and ABC/HRP system in normal and pathological human tissues were made. No immunoreaction was evidenced in normal tissues. The reactivity of 14F7 was detected in all adenocarcinomas of the stomach (12/12), colon (12/12), and pancreas (11/11). A finely granular immunorecognition in esophageal tumors (5/15), epidermoid carcinoma of the rectum (5/7), and basaloid carcinoma (4/5) of the latter as well as in hepatocellular carcinoma (13/14) was also observed. Our results are in agreement with the assumption that NeuGcGM3 ganglioside may be considered as target for passive and active immunotherapy in digestive system malignancies expressing this molecule.

## 1. Introduction

The digestive cancers are the first cause of mortality worldwide [1]. The most common malignancies of this system are epidermoid carcinomas derived from oral cavity, esophagus, and anal epithelium as well as adenocarcinomas, originated mainly in esophagus, stomach, and colorectal segment. Other neoplasms such as lymphomas, sarcomas, and neuroendocrine tumors can be considered relatively rare [2].

Despite of the significant advances in cancer therapy, the therapeutic options available for digestive tumors are insufficient. Particularly, ineffective treatments have been applied in patients bearing pancreas, stomach, and liver cancers. Patients with other malignancies such as colorectal tumors have a longer life expectancy than those with the before-mentioned cancers [3–5]. This fact promotes the development of alternative treatment strategies using target molecules. 

Gangliosides, sialic acid-containing glycosphingolipids, are considered attractive targets for cancer immunotherapy and diagnosis based on their higher presence in tumors as compared with normal tissues, as well as on their relevance in tumoral growth [6]. 

The molecular basis for the absence of N-Glycolyl neuraminic acid in human cells is due to a partial deletion in the gene that encodes CMP-Neu5Ac hydroxylase, the enzyme responsible for NeuGc biosynthesis [7]. However, the expression of N-Glycolylated gangliosides has been reported in several malignancies [8]. We reported the expression of N-Glycolyl GM3 (NeuGcGM3) ganglioside in breast tumors by chemical analysis [9]. Later we generated the 14F7 Mab highly specific against the NeuGcGM3 ganglioside that recognized breast infiltrating ductal carcinoma and melanomas by immunohistochemistry [10]. We also demonstrated the ability of 14F7 Mab labelled with 99mTc to recognize breast tumors *in vivo* by the radioimmunoscintigrafic technique [11]. 

The analysis of NeuGcGM3 expression in different human neoplasms could be useful in order to extend the assessment of this molecule as target for cancer immunotherapy. Here, we present the 14F7 Mab immunohistochemical recognition in different human normal and malignant tissues from digestive system, on both, gastrointestinal tract and annex glands.

## 2. Materials and Methods

### 2.1. Monoclonal Antibody

14F7 Mab (IgG1) a highly specific anti- NeuGcGM3 ganglioside antibody was generated by immunization of Balb/c mice with NeuGcGM3 hydrophobically conjugated with human very low-density lipoproteins (VLDL) adjuvated with Complete Freud adjuvant (CFA). This Mab was obtained by the hybridoma resulting of the fusion of spleen cells with mouse myeloma cell line P3X63Ag653 as described [10]. 

### 2.2. Tissue Specimens and Previous Processing

Sections of archival formalin-fixed and paraffin-embedded tissues from 72 primary human tumors from digestive system and 25 sections of normal tissues were evaluated by immunoperoxidase staining. 

### 2.3. Formalin-Fixed and Paraffin-Embedded Tissues

Routinely processed, formalin-fixed and paraffin-embedded archival tissue samples were taken from the pathology department of Dr. “Manuel Fajardo” General Teaching Hospital after it was approved by the clinical ethical committee. Five micrometer serial sections from each block were obtained in a micrometer and mounted on plus slides (Dako S2024, Carpinteria; USA). All sections were attached to the slide by heating in a 70°C oven for 1 h. Afterwards the slides were kept at room temperature until they were used.

The slides were dewaxed in xylene and rehydrated in graded ethanol series as usual and endogenous peroxidase activity was blocked with 0.03% hydrogen peroxide in absolute methanol for 30 minutes. All sections were rehydrated in distilled water for 10 minutes and rinsed with TBS, and then incubated with Dako blocking protein solution (Dako X0909, Carpinteria; USA) for 10 minutes at room temperature to reduce nonspecific background staining.

### 2.4. Immunohistochemical Staining

Subsequently, slides were placed in a humid chamber and incubated with the primary mouse anti-NeuGcGM3 ganglioside 14F7 Mab (10 *μ*g/mL) for 1 h at room temperature. Negative controls were performed substituting primary antibody for washing buffer (TBS) and breast infiltrating ductal carcinoma was used as positive control [10].

After two rinses in TBS, the tissues were incubated with a rabbit anti-mouse IgG policlonal antibody (Dako E0354, Carpinteria; USA) dilution 1 : 100 and with the avidin-biotin/peroxidase complex (Dako K0355, Carpinteria; USA) dilution 1 : 100 for 30 minutes each one. Among incubation the slides were washed with TBS 1X for 10 minutes. 

Enzymatic activity was visualized with DAB substrate chromogen solution (Dako K3465, Carpinteria; USA) and the samples were counterstained with Mayer's Hematoxylin (Dako S2020, Carpinteria; USA). Slides were dehydrated and coverslipped with a synthetic mounting medium.

### 2.5. Evaluation

The intensity of reaction of each patient tumor tissues was judged as negative (−), weak (+), moderate (++), and strong (+++), and we used a combination of this patterns to express intermediate levels of immunostaining. The percentage of tumor cells showing 14F7 Mab immunoreactivity was estimated using 10x optical microscopy fields selecting the most cellular regions of each section, and it was classified as 0 (negative to less than 5%), 1 (6–25%), 2 (26–50%) and 3 (more than 50%). The results in agreement with two different observers were considered as final. 

## 3. Results

### 3.1. Immunohistochemical Staining in Normal Tissues

The results of 14F7 Mab immunoreactivity on some formalin-fixed and paraffin-embedded normal tissues from digestive system (Stomach, Large intestine, Pancreas and Liver) are showed in Table 1. No immunorecognition with 14F7 Mab was observed, except for 3/12 (25%) of cases that showed a weak to moderate 14F7 Mab reaction in some normal glands located in peritumoral areas.

### 3.2. Immunohistochemical Staining in Neoplastic Tissues

The results of 14F7 Mab immunoreaction on some primary human tumors from gastrointestinal tract and annex glands are showed in Table 2. 

### 3.3. Gastrointestinal Tract

#### 3.3.1. Esophagus

**Figure 1:**
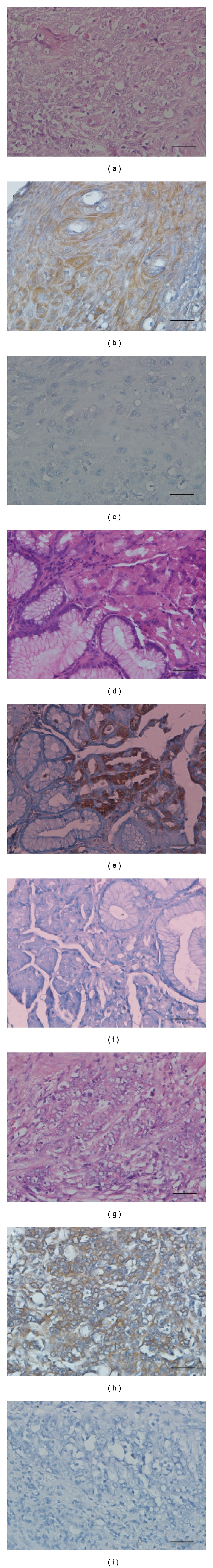
Hematoxylin and eosin staining of well-differentiated carcinoma of esophagus (a), adenocarcinoma of gastroesophageal junction (d), and gastric adenocarcinoma (g). Immunorecognition of 14F7 Mab in before-mentioned tumors (b, e, h). Note a moderate (finely granular) and strong reactivity located on plasmatic membrane and cytoplasm of malignant epithelial cells. For negative controls (c, f, i), tissues were stained as described in Section 2 without 14F7 Mab. No staining occurred in control sections. Black bar = 50 *μ*m.

Around 30 percent of esophageal tumors studied (5/15) were staining by 14F7 Mab (Table 2). Two different patterns of staining were observed in 2/11 tumor samples from the upper portion of the esophagus. One sample showed a weak to intense and heterogeneous reaction located mainly in cellular clusters (Data no shown), while the second pattern was observed as the same pattern of recognition of the lower portion of the esophagus. Moreover, both samples showed smaller percentage of positive cells than other tissues (Figure 1). The samples belonging to the lower portion of the esophagus exactly in the gastroesophageal junction exhibited a weak to intense, homogeneous and finely granular reaction located in cell membrane and cytoplasm in more than 75% of epithelial cells on 3/4 (75%) of cases (Figure 1). 

#### 3.3.2. Stomach

A moderate to intense immunoreaction with 14F7 Mab was observed in more than 95% of malignant cells from the glandular epithelium (12/12) of this organ. The immunoreaction not related to the grade of differentiation (*P* = .27 by CHI-SQUARE Test). The immunorecognition of this Mab was observed on membrane and cytoplasm of the above mentioned cells, although a membrane pattern was also evidenced (Figure 1). The staining was homogeneous and finely granular. Additionally, 3/12 (25%) of cases showed a 14F7 Mab reaction in some normal glands located in peritumoral areas. The pattern of staining was homogeneous and finely granular in 2/12 cases and heterogeneous in 1/12 cases.

#### 3.3.3. Colon

**Figure 2:**
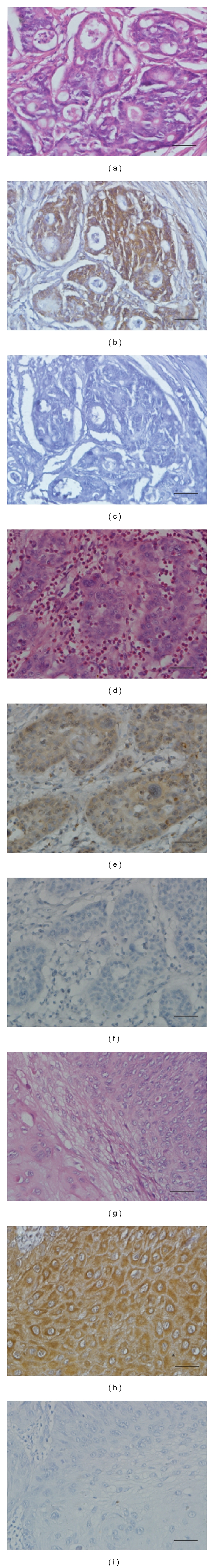
Hematoxylin and eosin staining of colonic adenocarcinoma (a), basaloid carcinoma (d), and epidermoid carcinoma (g) of the rectum. Strong immunostaining was detected on colonic adenocarcinoma (b) and epidermoid carcinoma of the rectum (h), while basaloid carcinoma of the rectum was weakly reactive (e) with 14F7 Mab. No staining occurred in all negative controls studied (c, f, i). Black bar = 50 *μ*m.

Colonic adenocarcinomas exhibited a homogeneous and finely granular reaction with 14F7 Mab in 12/12. The intensity of this reaction varied from weak to intense and was observed in more than 75% of malignant glandular cells. The staining was mainly located in the plasmatic membrane and also in the cytoplasm of the above mentioned cells. This pattern of expression was detected in 9/12 (75%) of studied cases (Figure 2). A heterogeneous immunostaining with 14F7 Mab in 3/12 (25%) cases was evidenced. 

#### 3.3.4. Rectum

Basaloid carcinoma showed a weak to intense reaction in more than 70% of neoplastic cells. This immunorecognition was located in plasmatic membrane and cytoplasm of these cells in 4/5 (80%) of cases (Figure 2). 

A moderate to intense reaction with 14F7 Mab was observed in 5/7 (71, 4%) of epidermoid carcinoma tested. This recognition was evidenced homogeneous and finely granular in more than 90% of tumor cells, and it was located in the plasmatic membrane and cytoplasm of these cells (Figure 2). 

### 3.4. Annex Glands

#### 3.4.1. Liver

**Figure 3:**
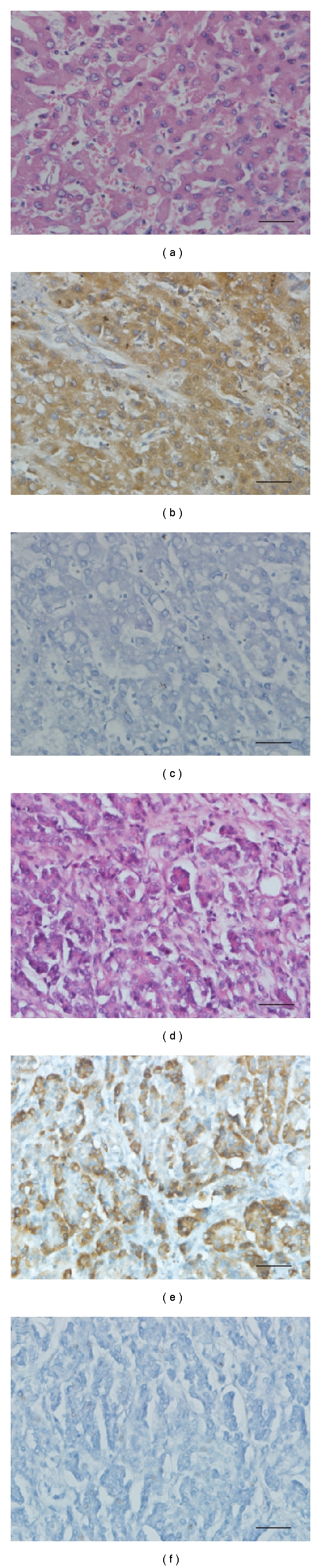
Hematoxylin and eosin staining of hepatocellular carcinoma (a) and pancreatic adenocarcinoma (d). See a strong and finely granular immunoreactivity of 14F7 Mab on both malignant hepatocytes (b) and glandular malignant cells derived from pancreas (e). The reaction was located on plasmatic membrane and cytoplasm. No staining occurred on negative controls sections (c and f, resp.). Black bar = 50 *μ*m.

A moderate to intense immunostaining with 14F7 Mab was observed in more than 90% of tumor cells in 13/14 (92, 8%) of hepatocellular carcinomas. The recognition of this Mab was located in membrane and cytoplasm of neoplastic hepatocytes showing a homogeneous and finely granular pattern (Figure 3). Just one case of hepatocellular carcinoma showed a weak to moderate reaction.

#### 3.4.2. Pancreas

The 14F7 Mab immunoreacted strongly with more than 80% of pancreatic neoplastic cells in the poorly differentiated adenocarcinomas tested (3/3). The staining was located mainly on the plasmatic membrane of malignant cells although the cytoplasm was also reactive. Well-differentiated adenocarcinomas showed a moderate to intense immunostaining located in the membrane and cytoplasm in more than 90% of tumor cells in 8/8 (Figure 3). Two patterns of recognition were evidenced in pancreas malignancies: the first, homogeneous and finely granular; the second, homogeneous and little grosser. The intensity of immunoreaction with 14F7 Mab was not related to the grade of differentiation (*P* = .72 by Fisher's Exact Test).

## 4. Discussion

The changes in carbohydrate pattern expression associated with the oncogenic transformation of the cells have been described for certain types of tumors [6, 12]. Usually neoplastic cells exhibit aberrant overexpression of gangliosides present or not in normal adult tissues becoming attractive targets for immunotherapy [13, 14]. 

Aberrant and elevated expression of gangliosides has been previously demonstrated on the surface of tumors derived from some stomach, pancreas, and large intestine as compared with cells of the normal tissues. Moreover, the majority of the studies of gangliosides expression in human malignancies have been restricted to N-Acetylated variant of sialic acid [6, 15–17]. 

N-Glycolyl neuraminic acids residues are considered not to be expressed in human normal tissues [18] due to the inactivation of the gene for CMP-Neu5Ac hydroxylase, the enzyme responsible for NeuGc biosynthesis [7]. However, small levels of expression have been found in some normal human tissues (e.g., epithelial cells and their secretions) [10, 19, 20]. The preferential expression in human malignancies have been related with the incorporation of N-Glycolyl neuraminic acids residues from dietary sources due to the altered and more accelerated metabolism of neoplastic cells [19, 20]. Others have also suggested an alternative pathway for the synthesis of Neu5Gc hydroxylase despite that normal human cells do not have a metabolic pathway for the production of N-Glycolyl neuraminic acids residues [21].

The antigenic determinant of Hangnutziu Deicher (HD) antigen is N-Glycolyl neuraminic acid [22]. Several studies with specific antibodies against HD antigen or anti- N-Glycolylated gangliosides antibodies have been reported in malignant tissues [9, 10, 23].

The 14F7 Mab recognition is strictly limited to N-Glycolyl function so it is able to differentiate between N-Glycolyl and N-Acetyl variants of GM3 ganglioside [10]. GM3 constitutes a normal component of the plasmatic membrane of normal human cells [10, 22, 24]. In our study we observed a cytoplasmatic reactivity of 14F7 Mab showing both finely and grosser granular pattern. These results could be supported by the dynamics intracellular movement of glycosphingolipids within the different subcellular compartments as well as their recycling from the plasmatic membrane to Golgi apparatus where they may be remodeled [25, 26].

Both pancreatic carcinoma and gastric adenocarcinoma confers one of the highest mortality rates in malignant human tumors [1]. Regardless the advances with some drugs, these neoplasms are two of the most lethal cancers and constitute an enormous challenge to clinicians and cancer scientists in order to diagnose and treat them. Here, we showed the reactivity of 14F7 Mab in all, differentiated or not, pancreatic carcinomas. A similar pattern was observed in gastric adenocarcinomas where 14F7 staining was not related to the grade of differentiation. These results could be relevant in order to consider NeuGcGM3 ganglioside as potential target in these two diseases, orphan of effective therapies. 

It is known that squamous cell and adenocarcinomas are responsible for more than 95 percent of all esophageal carcinomas [2]. In our study the majority of the squamous cell carcinoma from the upper esophagus was not stained with 14F7 Mab. However, a similar staining pattern in one of squamous cell carcinomas located in the gastroesophageal junction regarding gastric cancer was evidenced in our data, despite the differences in the cellular lineage that originated from the malignant neoplasm. Additionally, the adenocarcinomas of gastroesophageal junction showed a similar staining of the rest gastric tumors.

Zhang et al. [6] described the GM2 expression in gastric, pancreatic, and colonic tumoral biopsies by immunohistochemical studies using 696 Mab (IgM, anti-GM2 ganglioside antibody). While Diatlovitskaia et al. [16] reported a marked increase in the content of absolute gangliosides in gastric tumors regarding normal tissues, using biochemical and specific anti sera methods. Additionally, they found that GM3, GD3, and GM1 are the predominant gangliosides in most of the cases, whereas several polar components are minor ones. On the other hand, the expression of little amounts of GM3 lactone in gastric tumors, and its absence in normal tissues has been reported [17] as well as the increased amount of GM3 and GD3 gangliosides in colorectal and pancreatic carcinoma [15]. Recently, Distler et al. [27] published the expression of CD75s-1-ganglioside (IV(6)Neu5Ac-nLc4Cer) and the structurally closely related iso-CD75s-1-ganglioside (IV(3)Neu5Ac-nLc4Cer) by immunohistology of cryosections and semiquantitative thin layer chromatography (TLC) of tissue lipid extracts combined with mass spectrometry. These gangliosides have been considered as potential targets for adjuvant therapy in pancreatic cancer.

Here, we described the 14F7 (a highly specific IgG1 against NeuGcGM3) recognition in all colonic adenocarcinomas. More of 50% of the malignant cells were positive (moderate to intense immunostaining in 90% of tumors). While only 75% of rectum tumors were recognized by 14F7. The expression of N-Glycolyl neuraminic acid containing ganglioside in colonic cancers has been previously described by some authors [17, 22, 28, 29]. Higashi et al. [22] demonstrated the major presence of molecular species as NeuGcGM3, 4-O-acetyl NeuGcGM3, and NeuGcGM2 in the ganglioside fraction of some samples of colon tumors by TLC immunostaining, whereas no antigenic compound was detected in normal tissues. On the other hand, Hirabayashi et al. [28] confirmed the expression of NeuGcGM2 ganglioside in colonic cancers by two dimensional TLC immunostaining. Additionally, Watarai et al. [29] reported the expression of i-active ganglioside N-Glycolylated in colonic malignant tissues by immunohistochemical procedures using SHS-1 antibody specific against this antigen. 

Primary tumors of liver are the sixth most common cancer in the world and the third most common cause of death attributable to cancer. Most of these tumors are hepatocellular carcinomas [30]. Outstandingly, we obtained here a moderate to intense staining in more than 50% of positive cells in 13/14 cases of formalin-fixed and paraffin-embedded cancer tissues using 14F7 Mab. The detection of Hangnutziu Deicher antigen by flow cytometry in 3/15 patients with hepatocellular carcinoma was published by Koda et al. [23]. Later, the same group demonstrated the increase of NeuGc expression in hepatocellular carcinoma cells when tyramide signal amplification method was used. This technology was useful for the immunohistochemical detection of low levels of the HD antigens in cryosections of these tumors [31]. 

Previously we reported the NeuGcGM3 ganglioside biochemical measure by mass spectrometry and TLC immunostaining with monoclonal antibodies that recognized anti-N-Glycolylated epitope of sialic acid (P3) [8] or Hanganutziu Deicher [9]. Additionally, our group published the 14F7 Mab immunorecognition in primary melanomas and melanomas metastases as well as in human breast infiltrating ductal carcinoma [10, 32]. Later, the expression of NeuGcGM3 ganglioside in human breast cancer was supported by radioimmunoscintigraphic technique using the 14F7 Mab labeled with 99mTc where positive images indicating specific uptake of 99mTc-14F7 radioimmunoconjugate were obtained. This study permitted to visualize for the first time the recognition *“in vivo”* of 14F7 Mab in human breast primary tumors [11]. 

Moreover, in preclinical studies treatment with 14F7 Mab produced a strong antitumoral activity inducing cytotoxicity dependent and independent of complement [33, 34] suggesting that NeuGcGM3 should be considered as one attractive target for active and passive immunotherapy in digestive malignancies expressing this molecule.

The roles of NeuGcGM3 ganglioside on tumoral progression as well as its suppressor properties have been previous described [35, 36]. In addition, Labrada et al. [37] showed a consistent increase of NeuGcGM3 expression in the metastatic lesions as compared with primary tumors using the 3LL-D122 Lewis Lung Carcinoma spontaneous metastasis murine model. Our data and the relevance of NeuGcGM3 suggest that the expression of this molecule could be related with the aggressive behavior of digestive malignant cells. However, we did not distinguish changes in the level of NeuGcGM3 expression associated with histopathological grade in stomach and pancreas tumors. 

In summary, this study reports the immunohistochemical recognition of 14F7 Mab, a highly specific antibody against NeuGcGM3 ganglioside, in some malignant tissues derived from digestive system but not in normal sections. These data could support the possible use of NeuGcGM3 as target in digestive tumors. The mortality related to these cancers is expected to increase by 2020 [38]. NeuGcGM3 therapy targeting could be an effective approach to face that challenge. Clinical trials with NeuGcGM3/VSSP molecular cancer vaccine [39] and 1E10 ( an anti-idiotypic Mab that mimicry NeuGcGM3 ganglioside [40] are ongoing in metastatic breast cancer patients and advanced nonsmall cell lung cancer, respectively. 

## 5. Conclusions

The expression of NeuGcGM3 in some malignant tissues derived from digestive system but not in normal sections suggest that the expression of this ganglioside could be related to the aggressive behavior of malignant cells from digestive tumors. Our data could support the possible use of NeuGcGM3 as target for both active and passive immunotherapy of digestive system tumors expressing this molecule. 

## Figures and Tables

**Table 1:** Immunorecognition of 14F7 Mab on normal digestive system tissues.

Normal tissues	No. cases	Intensity	Positive cells
Paraffin-embedded			
Esophagus	ND	−	−
Stomach	3/8*	+/++	1
Large intestine	0/9*	−	0
Pancreas	0/4	−	0
Liver	0/4	−	0

ND: Not done, Intensity: − negative, + weak, ++ moderate, Positive cells: 0 (negative to less than 5%), 1 (6–25%), *samples from the normal tissue located in peritumoral area.

**Table 2:** Immunorecognition of 14F7 Mab on some primary human tumors from digestive system.

Histopathological type	No. cases (%)	Intensity range	Positive cells
Esophagus			
Upper portion			
Epidermoid Carcinoma	2/11 (18,2)	+/+++	1/2
Gastroesophageal junction			
Epidermoid Carcinoma	1/2 (50)	+++	3
Adenocarcinoma	2/2 (100)	+++	3
Stomach (adenocarcinoma)			
Well differentiated	7/7 (100)	++/+++	3
Moderate differentiated	2/2 (100)	+++	3
Poorly differentiated	3/3 (100)	++/+++	3
Large Intestine			
Adenocarcinoma	12/12 (100)	+/+++	3
Rectum			
Basaloid Carcinoma	4/5 (80)	+/+++	3
Epidermoid Carcinoma	5/7 (71,4)	++/+++	3
Pancreas (adenocarcinoma)			
Well differentiated	8/8 (100)	++/+++	3
Poorly differentiated	3/3 (100)	+++	3
Liver			
Hepatocellular carcinoma	13/14 (92,8)	++/+++	3

Intensity: − negative, + weak, ++ moderate, +++ intense. Positive cells: 0 (negative to less than 5%), 1 (6–25%), 2 (26–50%), and 3 (more than 50%).

## References

[1] Jemal A, Siegel R, Ward E, Murray T, Xu J, Thun MJ (2007). Cancer statistics, 2007. *Ca-A Cancer Journal for Clinicians*.

[2] Cotran RS, Kumar V, Collins T (1999). The gastrointestinal tract. *Robbins Pathologic Basis of Disease*.

[3] Bowles MJ, Benjamin IS (2001). ABC of the upper gasrointestinal tract: cancer of the stomach and pancreas. *British Medical Journal*.

[4] Saltz LB, Meropol NJ, Loehrer PJ, Needle MN, Kopit J, Mayer RJ (2004). Phase II trial of cetuximab in patients with refractory colorectal cancer that expresses the epidermal growth factor receptor. *Journal of Clinical Oncology*.

[5] Layke JC, Lopez PP (2006). Esophageal cancer: a review and update. *American Family Physician*.

[6] Zhang S, Cordon-Cardo C, Zhang HS (1997). Selection of tumor antigens as targets for immune attack using immunohistochemistry: I. Focus on gangliosides. *International Journal of Cancer*.

[7] Irie A, Koyamat S, Kozutsumi Y, Kawasaki T, Suzuki A (1998). The molecular basis for the absence of N-glycolylneuraminic acid in humans. *Journal of Biological Chemistry*.

[8] Vazquez AM, Alfonso M, Lanne B (1995). Generation of a murine monoclonal antibody specific for N-glycolylneuraminic acid-containing gangliosides that also recognizes sulfated glycolipids. *Hybridoma*.

[9] Marquina G, Waki H, Fernandez LE (1996). Gangliosides expressed in human breast cancer. *Cancer Research*.

[10] Carr A, Mullet A, Mazorra Z (2000). A mouse IgG monoclonal antibody specific for N-glycolyl GM3 ganglioside recognized breast and melanoma tumors. *Hybridoma*.

[11] Oliva JP, Valdés Z, Casacó A (2006). Clinical evidences of GM3 (NeuGc) ganglioside expression in human breast cancer using the 14F7 monoclonal antibody labelled with 99mTc. *Breast Cancer Research and Treatment*.

[12] Hakomori S (1989). Aberrant glycosylation in tumors and tumor-associated carbohydrate antigens. *Advances in Cancer Research*.

[13] Irie RF, Ravindranath MH, Borrebaeck CAK, Larrick GW (1990). Gangliosides as target for monoclonal antibodies therapy of cancer. *Therapeutic Monoclonal Antibodies*.

[14] Livingston PO, Zhang S, Lloyd KO (1997). Carbohydrate vaccines that induce antibodies against cancer. 1. Rationale. *Cancer Immunology Immunotherapy*.

[15] Fredman P, Nilsson O, Svennerholm L (1983). Colorectal carcinomas have a characteristic ganglioside pattern. *Medical Biology*.

[16] Diatlovitskaia EV, Tekieva EA, Lemenovskaia AF, Somova OG, Bergel’son LD (1991). Gangliosides GM3 and GD3 in human stomach and breast tumors. *Biokhimiya*.

[17] Tekieva EA, Diatlovitskaia EV (1993). Ganglioside lactones in human stomach and breast tumors. *Biokhimiya*.

[18] Kawai T, Kato A, Higashi H, Kato S, Naiki M (1991). Quantitative determination of N-glycolylneuraminic acid expression in human cancerous tissues and avian lymphoma cell lines as a tumor-associated sialic acid by gas chromatography-mass spectrometry. *Cancer Research*.

[19] Tangvoranuntakul P, Gagneux P, Diaz S (2003). Human uptake and incorporation of an immunogenic nonhuman dietary sialic acid. *Proceedings of the National Academy of Sciences of the United States of America*.

[20] Bardor M, Nguyen DH, Diaz S, Varki A (2005). Mechanism of uptake and incorporation of the non-human sialic acid N-glycolylneuraminic acid into human cells. *Journal of Biological Chemistry*.

[21] Malykh YN, Schauer R, Shaw L (2001). N-Glycolylneuraminic acid in human tumours. *Biochimie*.

[22] Higashi H, Naiki M, Matuo S, Okouchi K (1977). Antigen of ’serum sickness’ type of heterophile antibodies in human sera: identification as gangliosides with N-glycolylneuraminic acid. *Biochemical and Biophysical Research Communications*.

[23] Koda T, Shimosakoda T, Nishinaka S (1994). Diagnosis of hepatocellular carcinoma using chicken anti-monoclonal antibody—as tumor marker on the heterophilic hanganutziu-deicher antigen. *Gan To Kagaku Ryoho*.

[24] Nishimaki T, Kano K, Milgrom F (1979). Hanganutziu-Deicher antigen and antibody in pathologic sera and tissues. *Journal of Immunology*.

[25] Gordon CM, Lloyd KO (1994). Endocytosis and recycling of gangliosides in a human melanoma cell line: inhibitory effect of brefeldin A and monensin. *Archives of Biochemistry and Biophysics*.

[26] Gillard BK, Clement RG, Marcus DM (1998). Variations among cell lines in the synthesis of sphingolipids in de novo and recycling pathways. *Glycobiology*.

[27] Distler U, Souady J, Hülsewig M (2008). Tumor-associated CD75s- and iso-CD75s-gangliosides are potential targets for adjuvant therapy in pancreatic cancer. *Molecular Cancer Therapeutics*.

[28] Hirabayashi Y, Kasakura H, Matsumoto M (1987). Specific expression of unusual GM2 ganglioside with Hanganutziu-Deicher antigen activity on human colon cancers. *Japanese Journal of Cancer Research*.

[29] Watarai S, Kushi Y, Shigeto R (1995). Production of monoclonal antibodies directed to Hanganutziu-Deicher active gangliosides, N-glycolylneuraminic acid-containing gangliosides. *Journal of Biochemistry*.

[30] Blonski W, Reddy KR (2008). Hepatitis C virus infection and hepatocellular carcinoma. *Clinics in Liver Disease*.

[31] Koda T, Aosasa M, Asaoka H, Nakaba H, Matsuda H (2003). Application of tyramide signal amplification for detection of N-glycolylneuraminic acid in human hepatocellular carcinoma. *International Journal of Clinical Oncology*.

[32] Osorio M, Gracia E, Rodríguez E (2008). Heterophilic NeuGcGM3 ganglioside cancer vaccine in advanced melanoma patients: results of a Phase Ib/IIa study. *Cancer Biology and Therapy*.

[33] Carr A, Mesa C, Arango MDC, Vázquez AM, Fernández LE (2002). In vivo and in vitro anti-tumor effect of 14F7 monoclonal antibody. *Hybridoma and Hybridomics*.

[34] Roque-Navarro L, Chakrabandhu K, de León J (2008). Anti-ganglioside antibody-induced tumor cell death by loss of membrane integrity. *Molecular Cancer Therapeutics*.

[35] De Leòn J, Fernández A, Mesa C, Clavel M, Fernández LE (2006). Role of tumour-associated N-glycolylated variant of GM3 ganglioside in cancer progression: effect over CD4 expression on T cells. *Cancer Immunology, Immunotherapy*.

[36] de León J, Fernández A, Clavell M (2008). Differential influence of the tumour-specific non-human sialic acid containing GM3 ganglioside on CD4^+^CD25^−^ effector and naturally occurring CD4^+^CD25^+^ regulatory T cells function. *International Immunology*.

[37] Labrada M, Clavell M, Bebelagua Y (2010). Direct validation of NGcGM3 ganglioside as a new target for cancer immunotherapy. *Expert Opinion on Biological Therapy*.

[38] Eaton L (2003). World cancer rates set to double by 2020. *British Medical Journal*.

[39] Carr A, Rodríguez E, Arango MDC (2003). Immunotherapy of advanced breast cancer with a heterophilic ganglioside (NeuGcGM3) cancer vaccine. *Journal of Clinical Oncology*.

[40] Alfonso M, Díaz A, Hernández AM (2002). An anti-idiotype vaccine elicits a specific response to N-glycolyl sialic acid residues of glycoconjugates in melanoma patients. *Journal of Immunology*.

